# NMDA modulates oligodendrocyte differentiation of subventricular zone cells through PKC activation

**DOI:** 10.3389/fncel.2013.00261

**Published:** 2013-12-18

**Authors:** Fabio Cavaliere, Monica Benito-Muñoz, Mitradas Panicker, Carlos Matute

**Affiliations:** ^1^Departamento de Neurociencias, Universidad del País Vasco (UPV/EHU)Leioa, Spain; ^2^Achucarro Basque Center for Neuroscience, Universidad del País Vasco (UPV/EHU)Zamudio, Spain; ^3^Instituto de Salud Carlos III, Centro Investigación Biomédica en Red Enfermedades NeurodegenerativasLeioa, Spain; ^4^National Centre for Biological Sciences, UAS-GKVK CampusBangalore, India

**Keywords:** NMDA, PKC, NADPH oxidase, reactive oxygen species, multipotent cell differentiation

## Abstract

Multipotent cells from the juvenile subventricular zone (SVZ) possess the ability to differentiate into new neural cells. Depending on local signals, SVZ can generate new neurons, astrocytes, or oligodendrocytes. We previously demonstrated that activation of NMDA receptors in SVZ progenitors increases the rate of oligodendrocyte differentiation. Here we investigated the mechanisms involved in NMDA receptor-dependent differentiation. Using functional studies performed with the reporter gene luciferase we found that activation of NMDA receptor stimulates PKC. In turn, stimulation of PKC precedes the activation of NADPH oxidase (NOX) as demonstrated by translocation of the p67phox subunit to the cellular membrane. We propose that NOX2 is involved in the transduction of the signal from NMDA receptors through PKC activation as the inhibitor gp91 reduced their pro-differentiation effect. In addition, our data and that from other groups suggest that signaling through the NMDA receptor/PKC/NOX2 cascade generates ROS that activate the PI3/mTOR pathway and finally leads to the generation of new oligodendrocytes.

## INTRODUCTION

Extracellular glutamate is one of the most important neurotransmitters and neuromodulators of the CNS. It participates in different functions such as synaptic plasticity, LTP and learning, glia-neuron communication, or transduction of sensory input at the periphery and nociceptive pathway (for a review, see Fellin and Carmignoto, 2004; Carozzi et al., 2008; Niswender and Conn, 2010). In addition, at high concentrations glutamate can contribute to excitotoxicity and apoptotic cell death. Glutamate exerts its function through two different subfamily of receptors, metabotropic and ionotropic, which are expressed in neurons, astrocytes, and oligodendrocytes as well (Matute et al., 2006; Matute, 2011a,b), but less is known about its role in modulating adult neurogenesis and gliogenesis.

Extracellular glutamate activates various ionotropic receptors. Depending on their molecular structure and pharmacology, they can be divided in AMPA (GluA1–GluA4), kainate (GluK1–GluK5), NMDA (GluN1, GluN2A–GluN2D, GluN3A-B) receptor subtypes. The receptors are heteromers and form ion channels, with fast response times, and generate an overall increase in Ca^+^^+^ along with influx of Na^+^. All functional NMDA receptors have the GluN1 subunit associated with other subunits. Moreover, NMDA receptor activation requires the co-activation of glycine (Kleckner and Dingledine, 1988), and the receptors bearing GluN3 subunits bear more affinity for this amino acid (Yao and Mayer, 2006).

Excessive stimulation of ionotropic glutamate receptors can trigger excitotoxic cell death (Matute, 2011b). For this reason the pharmacology of ionotropic glutamate receptors is important to develop new drugs for the treatment of neurological disorders such as brain ischemia, epilepsy, and more recently demyelinating diseases (Kim et al., 2011).

Depending on the stage of maturation, oligodendrocyte precursor cells (OPCs) and mature oligodendrocytes possess various combinations of glutamate receptor subtypes. Metabotropic receptors are highly expressed in OPCs but less in mature oligodendrocytes. This also would explain the higher sensitivity of OPCs to hypoxia with respect to mature oligodendrocytes (Deng et al., 2004). All mGluRs are expressed in OPCs but only receptors in group I are able to protect OPCs from excitotoxic death, by preventing ROS generation (Deng et al., 2004; Luyt et al., 2006). In turn, activation of mGluR can counteract the effects of NMDA receptors in oligodendrocyte differentiation (Cavaliere et al., 2012).**

The exact role of NMDA receptors during oligodendrocyte maturation is unclear (Matute et al., 2006; Cavaliere et al., 2012). There is evidence suggesting that NMDA receptor signaling in oligodendrocyte progenitors is not required for oligodendrogenesis and myelination (De Biase et al., 2011). Thus, genetic deletion of structural GluN1 from OPCs does not result in changes in proliferation, differentiation, or myelination. More recently, we observed that strong activation of NMDA receptors in oligodendrocytes precursor cells derived from subventricular zone (SVZ) multipotent cells increases their differentiation and myelination rate *in vitro*. This data was confirmed using NMDA receptor antagonists or specific knockdown of GluN1 by RNA interference in OPCs which prevented differentiation induced by NMDA (Li et al., 2013).

Here we studied the intracellular pathway primed by NMDA receptor activation leading to oligodendrocyte differentiation in a culture of rat SVZ neurospheres. We observed that activation of NMDA receptors stimulates differentiation via PKC/NADPH oxidase (NOX)-dependent ROS generation.

## MATERIALS AND METHODS

### NEUROSPHERE CULTURE

Cultures were prepared from 4 to 7-day-old Sprague-Dawley rat pups. The SVZ was isolated and minced with a McIllwain tissue chopper. SVZ tissue from two to three brains was digested for 10 min at 37°C in 5 ml of trypsin/EDTA (Sigma, Madrid, Spain). Digestion was stopped by adding an equal volume of trypsin inhibitor and 0.01% DNAse I (both from Sigma, Madrid, Spain) for 5 min at room temperature. The cell suspension was centrifuged for 10 min at 600 × *g* and the pellet mechanically dissociated 25 times in NeuroCult medium (Stem Cell Inc., Grenoble, France) using a glass Pasteur pipette and 20 times using 1 ml pipette tips. The cells that remained in suspension were decanted and the single cell suspension counted using the Neubauer method. Cells were seeded in proliferation medium (NeuroCult with 10% neural stem cell factors from Stem Cell Inc., 2 mM glutamine, penicillin/streptomycin mix, 20 ng/ml EGF (Promega, Madrid, Spain), 10 ng/ml bFGF (Promega), 10 ng/ml PEDF (Millipore, Madrid, Spain) at a density of 10^4^ cells/cm^2^ and cultivated in suspension for 7 days at 37°C, 5% CO2. EGF, bFGF, and PEDF were added fresh every 2–3 days.

### OLIGODENDROCYTE DIFFERENTIATION

After 7 DIV (days *in vitro*), cells were aggregated as neurospheres. The neurospheres were maintained for 3 days in oligodendrocyte differentiation medium (ODM) composed of DMEM with 4.5 mg/ml glucose and sodium pyruvate (Gibco, Barcelona, Spain), SATO (100 μg/ml BSA, 100 μg/ml transferrin, 16 μg/ml putrescine, 40 ng/ml thyroxine, 30 ng/ml tri-iodothryronine, 60 ng/ml progesterone, 40 ng/ml selenium, all of from Sigma), 6.3 mg/ml *N*-acetyl-cysteine (Sigma), 0.5 mg/ml insulin (Sigma), 1 μg/ml CNTF (Peprotech, London, UK), and 10 μg/ml NT3 (Peprotech). This step was considered to be the pre-commitment stage before oligodendrocyte differentiation. After 3 DIV, floating neurospheres were allowed to attach to cover slips previously treated with poly-ornithine in ODM and differentiated for 1–10 DIV in the presence of different compounds (G0 6983 from Tocris, Madrid, Spain; gp91 from Anaspec, Liege, Belgium). Differentiation was evaluated by immunofluorescence as a ratio of myelin basic protein (MBP, from R&D System, Madrid, Spain; used at 1:1000) positive cells determined by immunofluorescence to total nuclei determined by staining with DAPI. All experiments with NMDA were performed in the presence of 100 μM glycine.

### ANIMAL CARE

All experiments were approved by the local Animal Care Committee of the University of Basque Country (Spain) Animal Ethics committee, as relevant, following European Communities Council Directive of 22 September 2010 (2010/63/EU). Every possible effort was made to minimize animal suffering and the number of animals used.

### IMMUNOCYTOCHEMISTRY

Cell cultures on cover slips were fixed in 4% paraformaldehyde and permeabilized with 0.05% Triton and 5% normal goat serum in phosphate-buffered saline (PBS). Cells were incubated with MBP primary antibody at 1:1000 dilution for 2 h at room temperature and then washed three times with 0.05% Triton in PBS. All secondary antibodies at 1:200 were added and incubated for 1 h in the dark at room temperature (Molecular Probes, Barcelona, Spain). After three washes with 0.05% Triton in PBS, cells cultures were stained for 1 min at room temperature with DAPI and further washed with PBS. Finally, cover slips were mounted with Glycergel (Dako, Barcelona, Spain) and examined by fluorescence using the Apotome system (Zeiss, Goettingen, Germany).

### mRNA EXTRACTION AND QUANTITATIVE RT-PCR

Total RNA from neurospheres (approximately 5000 neurospheres) after pre-differentiation was extracted with a commercial kit (Life technologies-Ambion, Madrid, Spain). The quality of the total RNA was determined by agarose gel electrophoresis and 1 μg was reverse transcribed at 60°C for 60 min using Superscript SSIII (Invitrogen, Madrid, Spain). A 2 μl aliquot of each mRNA were used for real-time quantitative PCR. Primers specific for rat NOX1, NOX2, and NOX3 were designed having the following sequence; NOX1fwd: TAC GAA GTG GCT GTA CTG GTT G, NOX1rev: CTC CCA AAG GAG GTT TTC TGT; NOX2fwd: GGT TCC AGT GCG TGT TGC T, NOX2rev: TCT TAT GGA AAG TAA GGT TCC TGT CC; NOX4fwd: GGA AGT CCA TTT GAG GAG TCA T, NOX4rev: TGG ATG TTC ACA AAG TCA GGT C. GAPDH and Hprt1 primers were used for normalization and the standard curves were determined using PrimerExpress software (Applied Biosystems, Madrid, Spain). Real-time quantitative PCR reactions were performed for 40 cycles at 60°C in a BioRad CFX96 amplification system (BioRad, Madrid, Spain).

### LUMINESCENT ASSAY FOR PKC ACTIVITY

Pre-differentiated neurospheres were disgregated with Accutase (Sigma) and transfected by electroporation (Amaxa-Lonza, Madrid, Spain) with 3 μg of the plasmid pAP1-LightSwitch (SwitchGear genomics, Menlo Park, CA, USA). Cells were lysed at different times in the presence of the substrate for luciferase Luminescence produced by the luciferase reaction (luciferin + ATP + luciferase → AMP+light) was quantified with a luminometer (Sinergy HT by Biotek, Potton, Bedfordshire, UK)

### STATISTICAL ANALYSIS

Data are presented as means ± SEM. Statistical analysis was carried out with the Student *t* test and, in all instances, at least a value of *p* < 0.05 was considered significant.

## RESULTS

**FIGURE 1:**
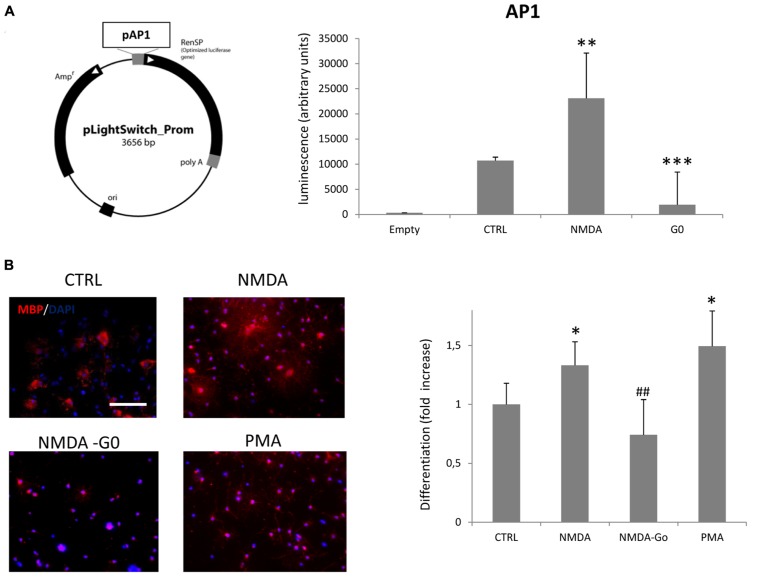
**Activation of NMDA receptors in neurospheres induces PKC activation and oligodendrocyte differentiation. (A)** Pre-differentiated neurospheres were dissociated and transfected with 3 μg of pAP1-LightSwitch. Cells were differentiated for 12 h in the presence of 100 μM NMDA or 100 nM G0 6893. Empty vector, without AP1 promoter, was transfected as a negative control. **(B)** Neurospheres were differentiated to oligodendrocyte for 3–5 days in the presence of 100 μM NMDA, 100 nM G0 6983, or 10 nM PMA. Cells were fixed, immunostained with anti-MBP (red) and counterstained with DAPI (blue; left panel). Differentiation was evaluated as the ratio between MBP positive cells vs. total cells counterstained with DAPI and expressed as a fold increase respect to the control (bar graph). Counts represent means ± SEM (*n* = 4 independent experiments, five fields in each). ***p < 0.001, **p < 0.01 and *p < 0.005 vs. CTRL, ##*p* < 0.01 vs. 100 μM NMDA. Scale bar = 100 μm.

Our previous results demonstrated that overstimulation of NMDA receptors of SVZ multipotent cells induced an increase of oligodendrocyte differentiation through NOX-dependent generation of ROS (Cavaliere et al., 2012). Here we hypothesize that NOX activation is induced by PKC activation. After proliferation and the pre-differentiation protocol (see Materials and Methods) we transfected pre-differentiated neurospheres with a plasmid (pLightSwitch) carrying the reporter gene luciferase under the control of the PKC-activated promoter AP1. In cells transfected with pLightSwitch, if PKC becomes activated, expression from the AP1 promoter increases which results in increased luciferase activity. PKC activity can therefore be registered by monitoring luminescence intensity after the reaction with the substrate, luciferin. On monitoring luciferase activity over a time course of 12, 24, and 72 h after differentiation, we detected a maximal PKC activity at 12 h post transfection (data not shown). At this time point the treatment of neurospheres with 100 μM NMDA during differentiation increased the basal level of PKC activity by 2.15-fold (**Figure 1A**), while NMDA treatment in the presence of the PKC inhibitor G0 6983 almost completely inhibited its activity. To confirm the involvement of PKC in NMDA mediated oligodendrocyte differentiation we counted the number of MBP^+^ cells vs. the total cells counterstained with DAPI in the presence of NMDA alone or in conjunction with G0 6983. As previously observed (Cavaliere et al., 2012), NMDA stimulation increased the differentiation rate by 30%, and this effect was blocked by the PKC inhibitor G0 6983. As a positive control of PKC-dependent differentiation we used the PKC activator phorbol 12-myristate 13-acetate (PMA), which increased the basal differentiation by nearly 50% (**Figure 1B**).

**FIGURE 2:**
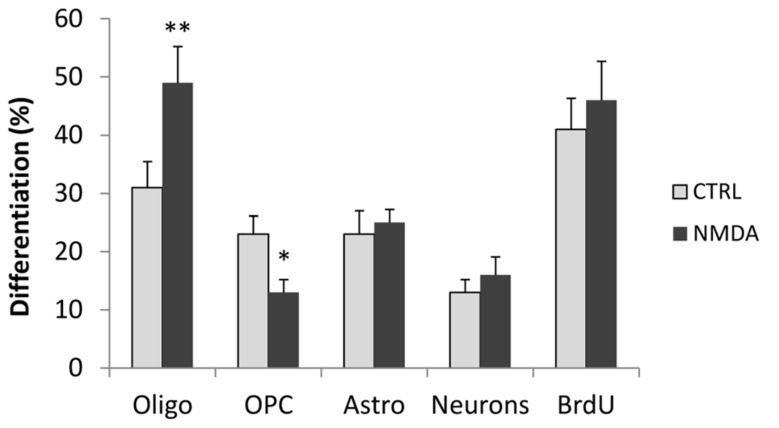
**Neurosphere cultures were differentiated for 3 days in the absence (CTRL) or presence of 100 μM NMDA.** After 2 days, proliferating cells were primed with 10 μM BrdU for 24 h, fixed and stained with markers to label mature oligodendrocyte (O4), OPC (PDGFR/O4), astrocytes (GFAP^+^/BrdU^-^), neurons (βIII tubulin), and total proliferating cells (BrdU). Results are expressed as a percentage of stained cells vs. total cells, counterstained with DAPI. Counts represent means ± SEM (*n* = 3 independent experiments, five fields in each). ^*^^*^p < 0.01, ^*^p < 0.05 vs. CTRL.

In addition, we evaluated the effect of NMDA stimulation on the differentiation of neurons and astrocytes, as well as on the proportion of OPCs that did not differentiate into mature oligodendrocytes. Cell cultures were stained after 3 days of differentiation with antibodies to PDGF receptor (PDGFR), to label only OPCs and with O4 that label both OPCs and mature oligodendrocytes. Mature oligodendrocytes were only positive for O4 whereas OPCs were positive for both markers. Treatment of cells with NMDA during differentiation induced an increase in the number of differentiated oligodendrocyte, but a significative reduction on the OPCs number (**Figure 2**), demonstrating the effect of NMDA on differentiation from immature to mature oligodendrocyte. To evaluate astrocyte differentiation we labeled the proliferating cells with 10 μM BrdU and stained the cultures with anti-GFAP and anti-BrdU antibodies. GFAP is expressed in both proliferating SVZ progenitor cells and in non-proliferating mature astrocytes.To quantify mature astrocytes we counted only GFAP^+^/BrdU^-^ cells. No significant differences were found between control and NMDA treated cells. Likewise, no differences were found on the number of differentiated neurons as assessed with βIII tubulin staining, or in the total number of proliferating cells (BrdU+ cells; **Figure 2**).

**FIGURE 3:**
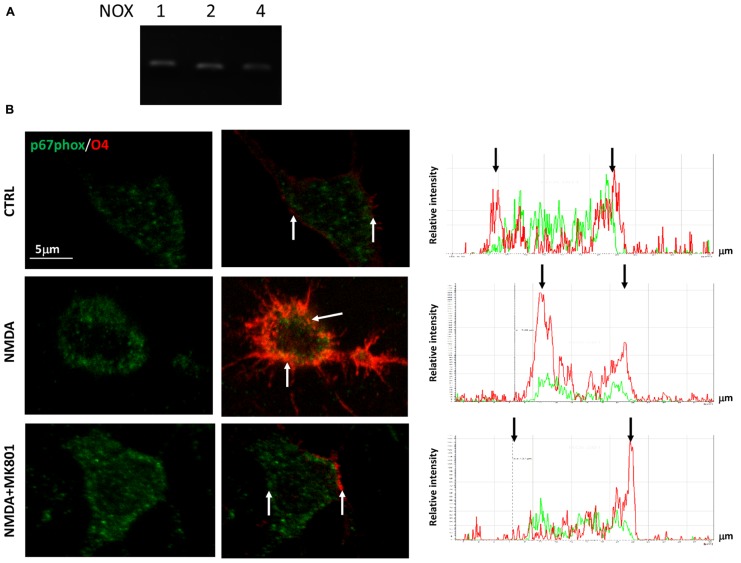
**(A)** cDNA from pre-differentiated neurospheres was analyzed by quantitative RT-PCR with primers for NOX1–2 and 4. Quantification was calculated as ΔΔDc_t_ relative values. Bar graphs represent the mean of four different experiments. **(B)** Neurospheres were differentiated to oligodendrocyte for three days in the presence of 100 μM NMDA and 50 μM MK801. Cells were fixed, immunostained with O4 (red) and p67phox (green). Bar = 5 μm. Analysis of colocalization shown on the right graphs was performed by the “Linescan” application of the LAS-AF software (Leica). Red and green peaks, belonging to O4 and p67 signal respectively, colocalized only in the NMDA treated cell, whereas in CTRL and NMDA+MK801 the p67 green signal is more dispersed along the cell body (black bar). White arrows point at cellular membrane sites corresponding to black arrows on the Linescan.

**FIGURE 4:**
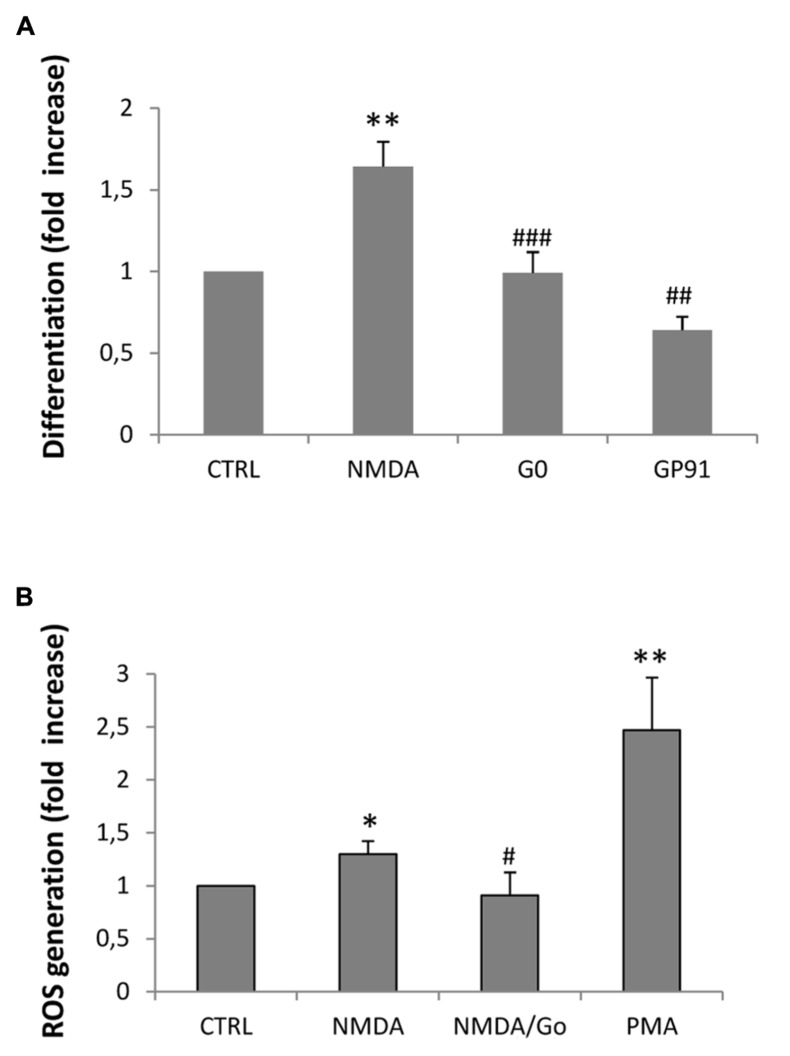
**(A)** Neurospheres were differentiated to oligodendrocyte for 3–5 days in the presence of 100 μM NMDA, 100 nM G0 6983, or 1mM gp91. Cells were fixed, immunostained with anti-MBP and counterstained with DAPI. Differentiation was calculated as a ratio between MBP positive cells vs. total cells counterstained with DAPI and expressed as a fold increase respect to the control. Counts represent means ± SEM (*n* = 4 independent experiments, five fields in each). ^**^p < 0.01 vs. CTRL, ^###^*p* < 0.001 and ^##^*p* < 0.01 vs. 1 μM NMDA **(B)** ROS generation was calculated by luminescence after 3 days of differentiation in the presence of 100 μM NMDA and the PKC inhibitor G0 6983. The PKC activator PMA was used as a control for PKC-dependent ROS generation. Counts represent means ± SEM (*n* = 4 independent experiments, five fields in each). ^*^p < 0.05 and ^**^p < 0.01 vs. CTRL, ^#^*p* < 0.05 vs. 1 μM NMDA.

In a previous study we demonstrated that NMDA-dependent oligodendrocyte differentiation occurred through the activation of NOX and the subsequent generation of intracellular ROS, which acted as a second messenger (Cavaliere et al., 2012). To establish the link between NMDA receptors, PKC and NOX activation, we first examined the expression profiles of the transcripts that encode the various NOXs expressed in the CNS (NOX1–2, 4) by quantitative RT-PCR. We found that all the NOXs examined are expressed in SVZ multipotent cells (**Figure 3A**). Furthermore, we observed that stimulation with NMDA (100 μM) during differentiation induced the traslocation of NOX subunit p67phox into the oligodendrocyte plasma membrane (Bedard and Krause, 2007), as demonstrated by confocal double immunofluorescence with O4 (**Figure 3B**). The use of the specific NMDA antagonist MK801 modulated the translocation, demonstrating the specificity of the NMDA effect. To get some insight as to which of the NOXs is involved in the modulation of oligodendrocyte differentiation we measured the differentiation rate after treatment of cells with NOX inhibitors. Gp91, which specifically inhibits NOX2, reverted the effect of NMDA during differentiation by 70% suggesting a pivotal role for NOX2 in oligodendrocyte differentiation (**Figure 4A**). The other general inhibitor, DPI, was toxic to the cells (data not shown). Finally, to further assess the involvement of the NMDA-PKC-NOX signaling cascade in modulating differentiation, we measured ROS generation during differentiation after stimulation with NMDA. We observed that activation of NMDA receptor induced a significant increase of ROS by 30% that is inhibited when the signaling in the NMDA-PKC-NOX pathway is blocked by the PKC inhibitor G06893 (**Figure 4B**).

## DISCUSSION

**FIGURE 5:**
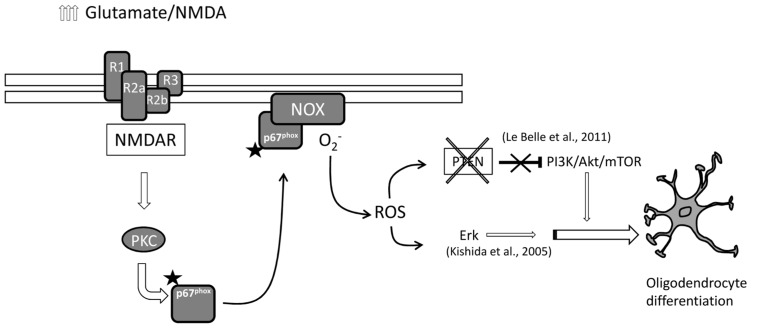
**Schematic representation of the hypothetical NMDA receptor-induced oligodendrocyte differentiation.** High levels of extracellular glutamate trigger NMDA receptor responses which activate PKC/NOX/p67 signaling. Activated NOX generates intracellular ROS that in parallel can set off PI3K/mTOR and/or ERK pathways to induce oligodendrocyte differentiation.

In this study we provide evidence supporting the idea that NMDA stimulates oligodendrocyte differentiation via activation of PKC (see also **Figure 5**). Oligodendrocyte differentiation from SVZ multipotent stem cells is modulated by fine tuned regulation of NMDA receptor subtypes, especially by the expression of NR3 subunit (Cavaliere et al., 2012). SVZ multipotent cells express high levels of NR3 with high NR3/NR1 ratio during proliferation but after stimulating oligodendrocyte differentiation, the same ratio reverts with higher NR1 expression respect to NR3 (Cavaliere et al., 2012). This stoichiometry reveals a dominant negative role of the NR3 subunit which has a low affinity for glutamate but higher affinity for glycine (Yao and Mayer, 2006) resulting in a lack of NMDA receptor function during proliferation. In SVZ oligodendrocytes differentiate from type C cells (transit amplifying cells). Depending on the culture condition or environmental constraints, type C cells can typically differentiate into neuroblasts, or alternatively into OPCs. In turn, we found that NMDA stimulates the final differentiation from OPCs into mature oligodendrocytes. The effect of NMDA during oligodendrocyte differentiation looks cell specific since no significant differences were observed in neuronal and astrocytic differentiation.

As expected, overactivation of NMDA receptors can lead to activation of PKC. When transfected with the luciferase reporter gene, under the control of the AP1 promoter, differentiating oligodendrocytes showed increased luciferase expression when treated with NMDA (**Figure 1A**). These data were corroborated also by a negative modulation of the PKC inhibitor G0 6983 on oligodendrocyte differentiation (**Figure 1B**). Extensive stimulation of NMDA receptors induced the generation of ROS via NOX stimulation through the activation of PKC, which ultimately resulted in hippocampal neuronal death (Brennan et al., 2009). Similarly, during oligodendrocyte differentiation, PKC activation by overstimulation of NMDA receptors induces NOX activation. SVZ multipotent cells express similar levels of mRNA encoding NOX1, NOX2, and NOX4 (**Figure 2A**) and selective inhibition of NOX activity by apocyanin and gp91, the specific inhibitor of NOX2 in the presence of NMDA (but not by Dpi; Cavaliere et al., 2012 and **Figure 3B**) suggested that only NOX2 can be involved in oligodendrocyte differentiation. Unlike hippocampal neurons, the generation of NOX-dependent ROS did not induce oligodendrocyte death but acted as a second messenger to stimulate progenitor differentiation as observed by others (Le Belle et al., 2011; Li et al., 2013). Furthermore treatment with NMDA during oligodendrocyte differentiation generates an increase in ROS production in parallel with differentiation, which is modulated by apocynin and gp91 (Cavaliere et al., 2012 and **Figure 3**) and the specific NMDA antagonist MK801.

## CONCLUSION

Results obtained from our group and others provide evidence for a mechanism by which mild excitotoxic insults promote oligodendrocyte differentiation derived from SVZ multipotent cells. These insults elevate the levels of ROS naturally generated in multipotent and pluripotent cells which act as a second messenger that induces oligodendrocyte differentiation through a proposed dual pathway (detailed in **Figure 4**). In one case, tumor suppressor protein PTEN is inactivated (Coant et al., 2010; Le Belle et al., 2011) and thus releases the blockade of the PI3/Akt/mTOR pathway (Li et al., 2013). In parallel, higher ROS levels can directly activate ERK pathway and stimulate differentiation as previously described (Kishida et al., 2005). In addition, a third mechanism has been recently suggested by which NMDA receptor activation regulates OPC migration and further differentiation by coupling to and activating the Tiam1/Rac1 pathway which is in turn is activated by PKC (Xiao et al., 2013).

In summary, our results highlight a novel signaling pathway directed by glutamate that drives oligodendrocyte development from SVZ, and that can favor remyelination in demyelinating diseases.

## Conflict of Interest Statement

The authors declare that the research was conducted in the absence of any commercial or financial relationships that could be construed as a potential conflict of interest.

## Author Contributions

Fabio Cavaliere, performed, conceived and designed the experiments; Monica Benito-Muñoz, performed the experiments; Mitradas Panicker, designed the experiments; Carlos Matute, designed the experiments.
